# ESGO/ESHRE/ESGE Guidelines for the fertility-sparing treatment of patients with endometrial carcinoma

**DOI:** 10.52054/FVVO.15.1.065

**Published:** 2023-03-31

**Authors:** A Rodolakis, G Scambia, F Planchamp, M Acien, A Di Spiezio Sardo, M Farrugia, M Grynberg, M Pakiž, K Pavlakis, N Vermeulen, G Zannoni, I Zapardiel, K.L. Tryde Macklon

**Affiliations:** Unit of Gynaecologic Oncology, Alexandra Hospital, National and Kapodistrian University of Athens School of Health Sciences, Athens, Greece; Fondazione Policlinico Universitario A. Gemelli IRCCS - Università Cattolica del Sacro Cuore, Roma, Italy; Clinical Research Unit, Institut Bergonie, Bordeaux, France; Obstetrics and Gynecology Department, San Juan University Hospital, Miguel Hernández University, Alicante, Spain; Gynecology and Obstetrics Unit, Department of Public Health, School of Medicine, University of Naples Federico II, Napoli, Campania, Italy; Spencer Private Hospitals, East Kent, UK; AP-HP, Department of Reproductive Medicine & Fertility Preservation, Hôpital Antoine-Béclère, Clamart, France; AP-HP, Department of Reproductive Medicine & Fertility Preservation, Hôpital Jean Verdier, Bondy, France; University Paris-Saclay, Saint-Aubin, France; Department for Gynecologic and Breast Oncology, University Medical Centre, Maribor, Slovenia; 1st Pathology Department, Alexandra Hospital, National and Kapodistrian University of Athens School of Health Sciences, Athens, Greece; Pathology Department, “IASO” Women’s Hospital, Athens, Greece; European Society of Human Reproduction and Embryology, Strombeek-Bever, Belgium; Department of Pathology, Dipartimento Scienze della Salute della Donna e del Bambino e di Sanità Pubblica, Fondazione Policlinico Universitario Agostino Gemelli IRCCS, Rome, Italy; Department of Gynecologic Oncology, La Paz University Hospital, Madrid, Spain; Fertility Department, Copenhagen University Hospital, Copenhagen, Denmark

**Keywords:** Uterine cancer, guideline, fertility preservation, endometrial carcinoma, oncofertility

## Abstract

**Background:**

The standard surgical treatment of endometrial carcinoma (EC) consisting of total hysterectomy with bilateral salpingo-oophorectomy drastically affects the quality of life of patients and creates a challenge for clinicians. Recent evidence-based guidelines of the European Society of Gynaecological Oncology (ESGO), the European SocieTy for Radiotherapy & Oncology (ESTRO) and the European Society of Pathology (ESP) provide comprehensive guidelines on all relevant issues of diagnosis and treatment in EC in a multidisciplinary setting. While also addressing work-up for fertility preservation treatments and the management and follow-up for fertility preservation, it was considered relevant to further extend the guidance on fertility sparing treatment.

**Objectives:**

To define recommendations for fertility-sparing treatment of patients with endometrial carcinoma.

**Materials and methods:**

ESGO/ESHRE/ESGE nominated an international multidisciplinary development group consisting of practicing clinicians and researchers who have demonstrated leadership and expertise in the care and research of EC (11 experts across Europe). To ensure that the guidelines are evidence-based, the literature published since 2016, identified from a systematic search was reviewed and critically appraised. In the absence of any clear scientific evidence, judgment was based on the professional experience and consensus of the development group. The guidelines are thus based on the best available evidence and expert agreement. Prior to publication, the guidelines were reviewed by 95 independent international practitioners in cancer care delivery and patient representatives.

**Results:**

The multidisciplinary development group formulated 48 recommendations for fertility-sparing treatment of patients with endometrial carcinoma in four sections: patient selection, tumour clinicopathological characteristics, treatment and special issues.

**Conclusions:**

These recommendations provide guidance to professionals caring for women with endometrial carcinoma, including but not limited to professionals in the field of gynaecological oncology, onco-fertility, reproductive surgery, endoscopy, conservative surgery, and histopathology, and will help towards a holistic and multidisciplinary approach for this challenging clinical scenario.

**What is new?:**

A collaboration was set up between the ESGO, ESHRE and ESGE, aiming to develop clinically relevant and evidence-based guidelines focusing on key aspects of fertility-sparing treatment in order to improve the quality of care for women with endometrial carcinoma across Europe and worldwide.

## Introduction

Endometrial carcinoma is the sixth most commonly diagnosed cancer in women worldwide, with increasing incidence in post-menopausal women (Sung et al., 2021). The estimated number of new cases of endometrial carcinoma in Europe in 2020 was 130 051 with 29 963 deaths, and the incidence has been rising with aging and increased obesity of the population (World Health Organization, 2020). Although not that common in pre-menopausal women, endometrial carcinoma, and its standard treatment of total hysterectomy with bilateral salpingo-oophorectomy drastically affects the quality of life of patients and creates a challenge for clinicians. Recent evidence-based guidelines of the European Society of Gynaecological Oncology (ESGO), the European SocieTy for Radiotherapy and Oncology (ESTRO), and the European Society of Pathology (ESP) provide comprehensive guidelines on all relevant issues of diagnosis and treatment in endometrial carcinoma in a multidisciplinary setting (Concin et al., 2021a; Concin et al., 2021b; Concin et al., 2021c). While addressing also work-up for fertility preservation treatments and the management and follow-up for fertility preservation, it was considered relevant to further extend the guidance on fertility-sparing treatment for endometrial carcinoma.

A collaboration was set up between the ESGO, the European Society of Human Reproduction and Embryology (ESHRE), and the European Society for Gynaecological Endoscopy (ESGE), aiming to develop clinically relevant and evidence-based guidelines focusing on key aspects of fertility- sparing treatment (patient selection, tumour clinicopathological characteristics, medical treatment, and special issues). These guidelines are intended for use by all health professionals who are involved in the fertility-sparing treatment of patients with endometrial carcinoma, across all allied disciplines. Even though our aim is to present the highest standard of evidence in an optimal fertility- sparing treatment, ESGO, ESHRE, and ESGE acknowledge that there will be broad variability in practices between the various centres worldwide and also significant differences in infrastructure, access to medical and surgical technology, and training, medicolegal, financial, and cultural aspects that will affect the implementation of any treatment guidelines.

### Responsibilities

These guidelines are a statement of evidence and consensus of the multidisciplinary development group based on their views and perspectives of currently accepted approaches for the fertility- sparing treatment of patients with endometrial carcinoma. Any clinician applying or consulting these guidelines is expected to use independent medical judgment in the context of individual clinical circumstances to determine any patient’s care or treatment. These guidelines make no warranties of any kind regarding their content, use, or application and the authors disclaim any responsibility for their application or use in any way.

## Materials and methods

**Figure 1:**
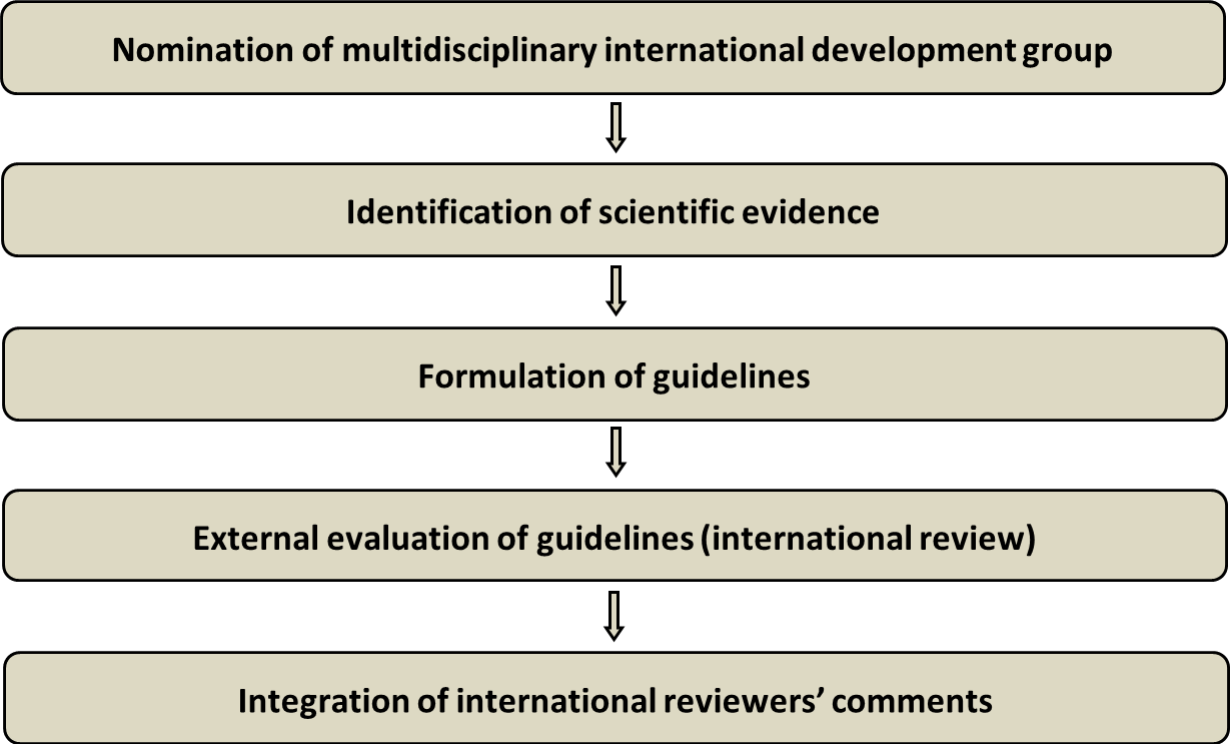
Development process.

The guidelines were developed using a five-step process as defined by the ESGO Guideline Committee standard operative procedures manual (Figure 1). The strengths of the process include creation of a multidisciplinary international development group, use of scientific evidence and international expert consensus to support the guidelines, and an international external review process (physicians and patients). This development process involved three meetings of the international development group, chaired by Professor Alexandros Rodolakis (National and Kapodistrian University of Athens, Greece, for ESGO), Dr Kirsten Tryde Macklon (University Hospital of Copenhagen, Denmark, for ESHRE), and Professor Giovanni Scambia (Fondazione Policlinico Universitario A Gemelli IRCCS, Rome, Italy, for ESGE).

**Figure 2:**
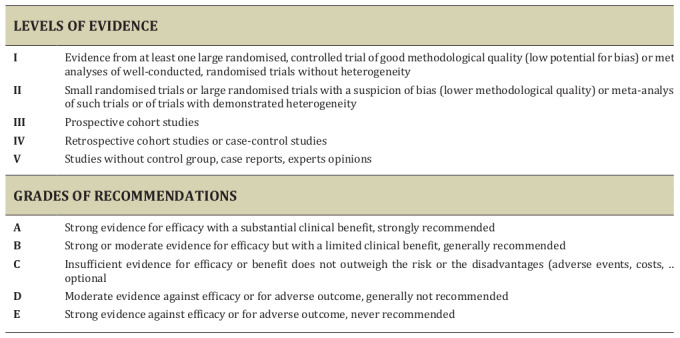
Levels of evidence and grades of recommendations.

ESGO/ESHRE/ESGE nominated practicing clinicians involved in the management of patients with endometrial carcinoma and who have demonstrated leadership through their expertise in clinical care and research, national and international engagement, and profile as well as dedication to the topics addressed to serve on the expert panel. The objective was to assemble a multidisciplinary development group. It was therefore essential for the validity and acceptability of the guidelines to include professionals from all relevant disciplines— that is, gynaecological oncology, onco-fertility, reproductive surgery, endoscopy, conservative surgery, and histopathology. To ensure that the statements were evidence-based, the current literature was reviewed and critically appraised. A systematic, unbiased literature review of relevant studies published between September 2016 and September 2021 was carried out using the Medline database (see online supplemental appendix 1 - https://fvvo.eu/assets/1091/Supplementary_data_1_-_identification_of_scientific_evidence.docx). The bibliography was also supplemented by additional older relevant references (if any). The literature search was limited to publications in English. Priority was given to high-quality systematic reviews, meta-analyses, and randomised controlled trials, but studies of lower levels of evidence were also evaluated. The search strategy excluded editorials, letters, and in vitro studies. The reference list of each identified article was reviewed for other potentially relevant articles. Based on the collected evidence and clinical expertise, the development group drafted guidelines for all the topics. These guidelines were discussed and retained if they were supported by sufficiently high-level scientific evidence and/or when a large consensus among experts was obtained. An adapted version of the Infectious Diseases Society of America- United States Public Health Service Grading System was used to define the level of evidence and grade of recommendation for each of the recommendations (Dykewicz, 2001) (Figure 2). In the absence of any clear scientific evidence, judgment was based on the professional experience and consensus of the development group.

ESGO/ESHRE/ESGE established a large multidisciplinary panel of practicing clinicians who provide care to patients with endometrial carcinoma to act as independent expert reviewers for the guidelines developed. These reviewers were selected according to their expertise and active involvement in clinical practice or research, while geographical balance ensured a global perspective. Patients with endometrial carcinoma were also included. The independent reviewers were asked to evaluate each recommendation according to its relevance and feasibility in clinical practice (only physicians), so that comprehensive quantitative and qualitative evaluations of the guidelines were completed. Patients were asked to evaluate qualitatively each recommendation (according to their experience, personal perceptions, etc). Evaluations of the external reviewers (n=95) were pooled and discussed by the international development group before finalizing the guidelines. The list of the 95 external reviewers is available in online supplemental appendix 2 - https://fvvo. eu/assets/1090/Supplementary_data_2_-_list_of_ the_95_external_reviewers.docx.

## Results and discussion

### Patient selection

Fertility-sparing treatment in endometrial carcinoma is an option for a subgroup of women who are selected based on thorough evaluation of reproductive potential. Fertility-sparing treatments should be exclusively applied in women with early-stage, non- metastatic disease. Implicit patient evaluation should take into consideration the reproductive potential and also risk factors that affect the potential for the patient to carry a pregnancy successfully, including the status of the uterus.

To date no literature exists on the reproductive potential specifically for women with endometrial carcinoma, although it must be assumed that the same markers of fertility apply to this group of patients as to any woman of fertile age. Markers of ovarian reserve, such as anti-Müllerian hormone, antral follicle count, and day 2–5 follicle- stimulating hormone levels as well as age and body mass index (BMI) of the patients can possibly all be used to estimate the ovarian function and the capacity of ovaries to produce mature oocytes after controlled ovarian stimulation. Patients with a diminished ovarian reserve might still benefit from fertility-sparing surgery, attempting a pregnancy with heterologous oocytes.

As with any woman seeking to become pregnant, age is a prognostic factor for success also in women with endometrial carcinoma. In a recent meta- analysis, it was found that the highest chance of achieving a live birth for women with endometrial carcinoma was in those younger than 35 years (live birth rate 30.7%). In studies including women up to age 40 years, a live birth rate of 23.0% was reported (Herrera Cappelletti et al., 2022).

Several lines of evidence indicate a strong relationship between weight and endometrial carcinoma. Indeed, maintaining a healthy weight or BMI, as well as weight loss through bariatric surgery or lifestyle changes in obese women, reduced the risk of endometrial carcinoma (Sundar et al., 2017). Being overweight or obese is considered to have a negative effect on fertility, conception, time to pregnancy, and pregnancy outcomes (Ribeiro et al., 2022). In overweight and obese women who have received endometrial carcinoma fertility-sparing therapy, weight loss could positively affect pregnancy rate and improve live birth rate (Chen et al., 2016; Gonthier et al., 2014; Obermair et al., 2020). A recent study showed that ≥5% weight loss increased pregnancy and live birth rates significantly in overweight and obese women (Zhang et al., 2021). Studies have demonstrated the positive effects of bariatric surgery for positive response rates to intra-uterine progestin, a reduction in systemic inflammation, and recruitment of immune cell types protective to the endometrium and a reduction in circulating biomarkers of insulin resistance (HbA1c and HOMA-IR (homeostasis model assessment of insulin resistance) and inflammation (hsCRP (high-sensitivity C-reactive protein) and IL-6 (interleukin 6) (Barr et al., 2021; MacKintosh et al., 2019; Naqvi et al., 2022).

Polycystic ovary syndrome is an endocrine system disorder among women of reproductive age represented by polycystic ovarian morphology with abnormal uterine bleeding. It is known as one of the causes of endometrial carcinoma, owing to prolonged exposure to oestrogen as well as persistent progesterone deficiency (Hardiman et al., 2003; Shao et al., 2014). Polycystic ovary syndrome is frequently found among patients with endometrial carcinoma who are under 35 years of age. These patients are more often obese, insulin-resistant, or diagnosed with more advanced disease (Okamura et al., 2017). Insulin resistance, a condition with hyperinsulinemia and hyperglycaemia due to the inability of muscle, liver, and fat cells to take up and store glucose sufficiently, is often seen in obese patients and can in the worst cases lead to type 2 diabetes. Women with polycystic ovary syndrome and endometrial carcinoma have been found to more often fail to respond to medroxyprogesterone acetate therapy (Okamura et al., 2017). Associated abnormalities, such as obesity, nulliparity, infertility, and diabetes, can all independently act as risk factors for endometrial carcinoma. Obesity and polycystic ovary syndrome additively contribute to the evolution of metabolic syndrome. Polycystic ovarian morphology (not necessarily polycystic ovary syndrome) may be a prognostic factor in patients with endometrial carcinoma who achieved complete remission after fertility-sparing therapy with progestin, independently of BMI (Fukui et al., 2017). Because of the risks associated with metabolic syndrome, it is important that women with polycystic ovary syndrome are aware of the positive effects of lifestyle changes and medical treatment to reduce their risk of cardiovascular disease and type 2 diabetes.

Lynch syndrome is associated with the development of endometrial carcinoma, often with an earlier age at onset, together with a detectable and treatable pre-malignant or early malignant stage (Dominguez-Valentin et al., 2020). With regards to fertility-sparing treatment, there is no consensus as to whether patients with endometrial carcinoma and Lynch syndrome could be considered as appropriate candidates, since there is no evidence on the safety of the conservative approach in this population (La Russa et al., 2018). Recently, a systematic review evaluating the potential prognostic factors of patients with early-stage endometrial carcinoma and complex atypical endometrial hyperplasia who received fertility-sparing treatment was published, but among the 1099 patients only nine (0.8%) had a family history of Lynch syndrome (Li et al., 2019). Outcomes of women treated with this conservative approach are good, even if fatalities following this treatment have been described (Ferrandina et al., 2005; Kothari et al., 2008).

Considering the specificities of Lynch syndrome and its association with other malignancies, some points should be taken into account on decision- making, in particular:

1. Younger age of endometrial carcinoma diagnosis and probably higher risk of disease progression.
2. Risk of synchronous ovarian cancer, which represents the third most common cancer in women with this syndrome (Nakamura et al., 2014).
3. Different molecular mechanisms cause the disease, and it is not clear if hormonal therapy can be effective. Indeed, in this syndrome, lesions are caused by genetic mutations, and the molecular mechanisms involved in the disease appear different from those of sporadic cancers (Corrado et al., 2021). However, patients with Lynch syndrome may at the same time also have a hyper-estrogenic state, that could be the cause of the endometrial carcinoma and that could potentially be treated with progestins.
4. Resistances to conservative treatment and recurrences are more common in mismatch repair-deficient patients (Chung et al., 2021; Raffone et al., 2021; Zakhour et al., 2017). In this case hysteroscopic resection has been described as an option for improving outcome (Chung et al., 2021).
  No data about the difference in management of endometrial carcinoma and complex endometrial hyperplasia are available—in particular, in the Lynch syndrome population.

#### Recommendations

##### General Recommendations

- Patients with a pregnancy wish should be referred to specialised care, especially those with genetic syndrome (Level of evidence V, grade A).
- Joint care and counselling with a multidisciplinary team of at least gynaecologic oncologists, fertility specialists, pathologists, and radiologists should be proposed to all patients with a pregnancy wish (Level of evidence V, grade A).

##### Reproductive Potential

- No recommendations can be given based on the literature. However, evaluation of the reproductive potential and consultation with fertility specialists should be performed prior to fertility-sparing treatment (Level of evidence V, grade B).

##### Age-specific Age Limits

- Women should be counselled about their reduced chances of achieving a live birth with their own gametes with increased age (Level of evidence II, grade A).

##### Health status, Obesity

- Following fertility-sparing therapy for endometrial carcinoma, weight loss in overweight and obese women or maintaining a healthy BMI is important for improving the chances of pregnancy (natural or after assisted reproductive technologies) and live birth. Therefore, weight loss in overweight and obese women or maintaining a healthy BMI after fertility-sparing treatment is strongly suggested as soon as possible (Level of evidence II, grade A).

##### Lynch Syndrome

- The presence of any concurrent/metachronous cancer should be determined (Level of evidence II, grade A).
- Patients should be informed about the higher risk of persistence/recurrence as compared with other patients (Level of evidence II, grade A).
- Fertility-sparing treatment in women with Lynch syndrome should be discussed on a case-by- case basis (Level of evidence II, grade A).

### Tumour clinicopathological characteristics

Pathological diagnosis of endometrial hyperplasia and endometrial carcinoma is of critical importance for optimal risk stratification and treatment decisions; therefore, diagnostic errors may strongly influence patient outcome. It was shown that using a World Health Organization (WHO) two- tier classification with two diagnostic categories, hyperplasia without atypia and endometrial hyperplasia/endometrioid intra-epithelial neoplasia, improved reproducibility (Bergeron et al., 1999; Kendall et al., 1998; Ordi et al., 2014; Trimble et al., 2006). The distinction between atypical endometrial hyperplasia and well-differentiated endometrial carcinoma showed poor intra-observer and inter-observer agreement (Grevenkamp et al., 2017). Moreover, poor inter-observer agreement exists when evaluating the grade of endometrial carcinomas specifically in curettage material (Lax et al., 2000; Scholten et al., 2004). Three additional retrospective single-institutional studies demonstrated a poor correlation between pre- operative endometrial sampling and final diagnosis (Garcia et al., 2017; Lago et al., 2018; Piotto et al., 2020). Although the International Society of Gynaecological Pathologists recommends the use of a binary grading system by combining grades 1 and 2 into a single low-grade category which reduces the degree of disagreement, for patients desiring a fertility-sparing treatment approach, it will continue to be necessary to distinguish grades 1 and 2 (Soslow et al., 2019).

Endometrial sampling has suboptimal accuracy in predicting the tumour grade compared with the final surgical specimen, especially in early- stage endometrial carcinoma in low/intermediate grade tumours (G1–G2). Therefore, as suggested in a multicentre prospective study on fertility- preserving surgery in endometrial carcinoma, a second opinion from an expert pathologist is important to minimise risk associated with preserving the uterus (Ushijima et al., 2007).

Recent publications advocate the use of the immunohistochemical evaluation of several biomarkers such as PTEN, PAX2, ARID1A or β-catenin in order to detect endometrial hyperplasia/ endometrioid intra-epithelial neoplasia, increasing thereafter inter-observer agreement. Yet, the use of the above markers for diagnostic purposes is still debated (Ayhan et al., 2015; Mutter et al., 2014; Sanderson et al., 2017; Yen et al., 2018).

The differentiation of endometrial carcinoma is the most important predictor of stage and response to treatment with progestins. Women with grade 1 stage IA endometrioid endometrial carcinoma (without myometrial invasion) seem to have a greater chance of responding to treatment with progestins, whereas the likelihood of presenting with advanced disease in the future is really low. In the available literature there are few reported cases of conservative treatment of grade 2 stage IA endometrial carcinoma. In a multicentre worldwide project endorsed by the Gynaecologic Cancer Intergroup, among 23 patients with grade 2 stage IA endometrioid endometrial carcinoma treated by hysteroscopic resection plus progestin, 17 patients showed complete response. The recurrence rate was 41.1% (Falcone et al., 2020). Five young women with grade 2 stage IA endometrial adenocarcinoma who wished to preserve fertility treated with combined oral medroxyprogesterone acetate/levonorgestrel-intra-uterine device showed a complete response in three of the five cases, with partial response in the other two patients (Hwang et al., 2017). Among four patients with grade 2 stage IA endometrial carcinoma treated with oral megestrol acetate (160 mg per day), with metformin (500 mg, three times a day) in cases of metabolic syndrome, 75% (3/4) of the patients had a complete response; one of whom relapsed and achieved again complete response and a fourth patient who had myometrial invasion during fertility-sparing treatment (Shan et al., 2021). Of eight patients with grade 2 presumed stage IA endometrioid adenocarcinoma who underwent fertility-sparing treatment, complete response was found in seven of the eight cases, with three developing a recurrence and were treated with second-line fertility-sparing therapy (Yu et al., 2020).

The cornerstone investigation for the diagnosis of endometrial carcinoma is an endometrial biopsy. Several methods to obtain endometrial tissue samples are in use, such as curettage techniques using a Pipelle, Novak, Vabra, or dilation and curettage using metal sharp curettes as well as hysteroscopic guided endometrial biopsy (Di Spiezio Sardo et al., 2020; Narice et al., 2018). Dilation and curettage has long been considered the standard method to obtain a histological diagnosis and despite its many deficiencies is still preferred by many authors (Rodolakis et al., 2015). Falcone et al showed that, compared with Pipelle biopsy, dilation and curettage is associated with the lowest rate (<10%) of histological under-grading and correlates better with the histological result of the final specimen (Auclair et al., 2019; Falcone et al., 2017). Several other studies dispute this and argue that a blind approach will sample less than 50% of the endometrial cavity. Consequently, nearly 10% of endometrial lesions could be missed—in particular, focal abnormalities, with a high percentage of false- negative results (Bettocchi et al., 2001; Goldstein, 2010). It is suggested that blind techniques should no longer be offered to obtain endometrial histology and a visually oriented hysteroscopic approach to diagnose endometrial carcinomas should be favoured (Ramshaw and Narayansingh, 2019).

**Figure 3:**
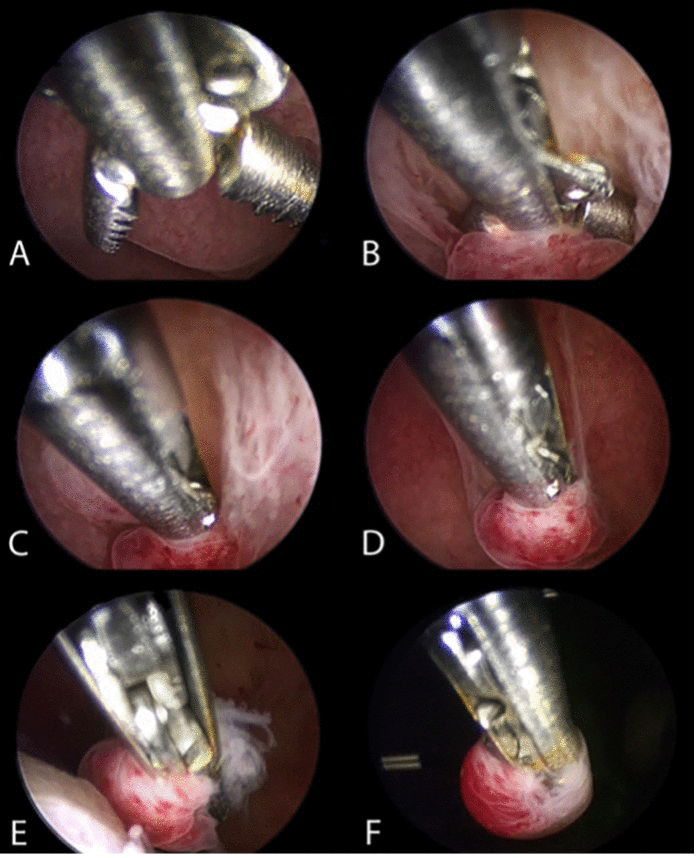
Hysteroscopic endometrial biopsy with “grasp technique” (sequentially ordered from A to F).

Over the past 25 years, hysteroscopy and directed endometrial biopsy has been recognised as the gold standard in diagnosing endometrial malignancy. The endometrial biopsy with ‘grasp’ technique has replaced the traditional hysteroscopic ‘punch’ biopsy, as it allows removal of larger portion of endometrial tissue. This technique achieves a high concordance of histologic type and tumour grade, especially in the presence of an endometrioid-type tumour (Auclair et al., 2019) (Figure 3). Once the area to biopsy has been identified, the alligator forceps is positioned with the jaws opened at the level of the endometrium to be sampled (Figure 3A). Next, the jaws are dragged across the tissue for about 0.5–1 cm (Figure 3B). At this point, the jaws are closed, grasping the piece of tissue to be examined (Figure 3C, D), which is then retrieved— together with the hysteroscope—from the uterine cavity, without retracting the tip of the forceps into the operating channel of the hysteroscope (Figure 3E, F).

Where the area to be biopsied is noted to be hypotrophic/atrophic, a different technique is more appropriate. Using a bipolar electrode or 5 Fr scissors, precise cuts can be made to collect adequate tissue samples, which are then removed with the grasping forceps. Another option can be the use of an intra-uterine tissue removal device, which allows collection of a larger amount of tissue, or of a 15 Fr bipolar office resectoscope, with a cutting loop, which allows tissue to be collected also from the subendometrial layer, when needed.

A meta-analysis of 65 studies on the accuracy of hysteroscopy in the diagnosis of endometrial carcinoma including 26 346 women (29% post- menopausal), assessed the diagnostic accuracy of hysteroscopy for the detection of endometrial carcinoma and hyperplasia (Clark et al., 2002). The overall sensitivity of hysteroscopy was 86.4% with a specificity of 99.2% for the detection of endometrial carcinoma (Clark et al., 2002). A meta-analysis evaluated the diagnostic accuracy of endometrial biopsy performed under direct hysteroscopic visualization versus blind or hysteroscopic oriented for diagnosis of endometrial pathology (Di Spiezio Sardo et al., 2022). Studies included a total of 1470 women and showed that hysteroscopic guided endometrial biopsy is more accurate for the diagnosis of endometrial pathology than blind or hysteroscopic oriented biopsy (Di Spiezio Sardo et al., 2022).

Whether hysteroscopy might increase the dissemination of tumour cells into the peritoneal cavity is an old debate; actually, the possible spread of malignant endometrial cells into the peritoneal cavity following diagnostic hysteroscopy has been shown not to alter tumour staging and has not been shown to adversely affect the patient’s prognosis (Chang et al., 2011). Tissue removal devices also do not result in increased dissemination of malignant cells into the peritoneal cavity when used as an initial biopsy method in the diagnosis of endometrial carcinoma and are not associated with surgical upstaging of patients compared with conventional endometrial biopsy methods (Kelly et al., 2021). The International Federation of Gynecology and Obstetrics (FIGO) staging system states that the confirmed diagnosis of a positive peritoneal washing does not alter the tumour stage and is recorded separately from the report issued on the staging itself (Amant et al., 2018).

The absence of myometrial invasion should be determined before making the decision to proceed with the fertility-sparing approach. The great majority of published trials are focused on evaluating performance of different imaging modalities on assessment of deep myometrial invasion. The methodology and statistical analysis are therefore set to estimate sensitivity and specificity to discriminate 50% of myometrial invasion. There are no specific data for discriminating between no myometrial invasion and shallow myometrial invasion. So, the performance of transvaginal ultrasound or MRI for determining absence of myometrial invasion or shallow myometrial invasion is being extrapolated from the data about diagnosing deep myometrial invasion.

Myometrial invasion can be evaluated using different techniques, including transvaginal ultrasound and pelvic MRI (Alcazar et al., 2015; Bi et al., 2020; Christensen et al., 2016; Costas et al., 2022; Fruhauf et al., 2017; Kim et al., 1995; Manfredi et al., 2004). Transvaginal ultrasound and pelvic MRI show comparable diagnostic performances in assessing myometrial invasion and cervical stromal invasion in early endometrial carcinoma. A systematic review and meta-analysis showed the sensitivity and specificity of transvaginal ultrasound for diagnosing deep myometrial invasion are 75% and 82%, respectively. The sensitivity and specificity for MRI according to the same review is 83% and 82%, respectively, without any statistical differences observed (Alcazar et al., 2017). The vast majority of trials report similar results, with sensitivity and specificity ranging between 75% and 84% for transvaginal ultrasound and between 82% and 90% for MRI (for diagnosing deep myometrial invasion). The sensitivity and specificity for cervical stromal invasion ranges between 69% and 82% and between 93% and 96%, both for transvaginal ultrasound and MRI (Alcazar et al., 2019; Bi et al., 2021a; Bi et al., 2021b; Bi et al., 2020; Capozzi et al., 2021; Chen et al., 2021; Christensen et al., 2016; Costas et al., 2022; Cubo-Abert et al., 2021; Gaston et al., 2022; Gil et al., 2019; Goel et al., 2019; Guo et al., 2017; Jonsdottir et al., 2021; Lin et al., 2017; Masroor et al., 2018; Nagar et al., 2021; Pineda et al., 2016; Rodriguez- Trujillo et al., 2016; Sanchez et al., 2019; Sobocan et al., 2021; Tong et al., 2021; Wang et al., 2021; Yang et al., 2021; Yildirim et al., 2018).

Subjective assessment of myometrial invasion yields the highest diagnostic accuracy (overall accuracy of 75.7%) compared with objective methods, such as deepest invasion/normal myometrium ratio (overall accuracy of 67.3 %) or tumour/uterine anteroposterior diameter ratio (overall accuracy of 68.1%) (Fruhauf et al., 2017). The diagnostic accuracies of transvaginal ultrasound and MRI are the highest when performed by expert practitioners. The advantage of MRI over transvaginal ultrasound is mainly the contribution of MRI for assessing extra-uterine disease (i.e., lymph node assessment).

The probability of extra-uterine disease or lymph node involvement for early-stage, low-risk endometrial carcinoma is extremely low. However, the basic clinical-radiologic staging should be performed (as surgical staging is not possible in a fertility-sparing approach). Chest radiology, either CT scan or a plain X-ray examination, should be performed in all women with endometrial carcinoma to exclude pulmonary spread (Sundar et al., 2017). Abdominal ultrasound (US) or CT can be used for evaluating the spread to abdominal organs. Lymph nodes can be assessed using CT, MRI or positron emission tomography (PET)-CT. MRI is a good diagnostic tool for detecting pelvic or para-aortic lymph nodes with a low to moderate sensitivity but a high specificity. PET-CT shows the highest specificity but a moderate sensitivity for detecting lymph node metastasis. The choice of the diagnostic tool (US, CT, PET-CT, MRI) should be made individually according to the patient’s characteristics and imaging accessibility (Bi et al., 2020; Bus et al., 2021; Xu et al., 2019; Yang et al., 2019). Synchronous or metastatic ovarian cancer occurs in 5–29% of patients with endometrial carcinoma, and younger women <45 years of age are five times more likely to have synchronous ovarian cancer than women aged >45 years (Obermair et al., 2020). However, in women with low-risk disease (no myometrial invasion, grade 1 endometrioid histology, normal looking ovaries) no cases of ovarian cancer were detected (Knez et al., 2021).Adnexal involvement can be identified using pelvic MRI or transvaginal ultrasound (Guillon et al., 2019; Obermair et al., 2020). There are no data about systematic use of sentinel lymph node biopsy in a fertility-sparing setting. The probability of lymph node involvement in low- risk endometrial carcinoma without myometrial invasion is extremely low and therefore sentinel lymph node biopsy is not recommended in a fertility-sparing approach.

#### Recommendations

##### Review of initial pathology by an experienced histopathologist

- A request for a second opinion by an experienced histopathologist is recommended if fertility-sparing treatment is considered (Level of evidence III, grade A).
- The G1, G2, G3 grading system is recommended. The binary grading system for endometrial carcinoma should not be used for these patients (Level of evidence III, grade A).
- The use of immunohistochemistry (PTEN, ARID1A, etc) for the evaluation of several biomarkers is not recommended for diagnostic purposes (Level of evidence IV, grade D).

##### Differentiation of the Tumour

- Fertility-sparing treatment is considered for endometrioid patients with endometrial carcinoma with grade 1, stage IA without myometrial invasion and without risk factors (Level of evidence V, grade A).
- Evidence for grade 2 endometrioid endometrial carcinoma is limited. Therefore fertility-sparing treatment should be discussed on a case-by-case basis (Level of evidence IV, grade C).

##### Establishing a Reliable Histopathology

- Hysteroscopic guided endometrial biopsy is preferred over blind biopsy for confirming diagnosis of endometrial carcinoma (Level of evidence III, grade A).

##### Myometrial Invasion

- Pre-operative assessment of myometrial invasion in patients with endometrial carcinoma should be performed using MRI or transvaginal ultrasound by a specialised radiologist/sonographer. Standardised high-quality protocols for MRI should be used to reach the highest possible accuracy (Level of evidence III, grade A).
- CT should not be used for pre-operative assessment of myometrial invasion in patients with endometrial carcinoma (Level of evidence III, grade A).

##### Exclude Extra-Uterine Disease/Synchronous or metastatic

- MRI or CT scan is recommended for detecting pelvic or para-aortic lymph nodes and distant metastases (Level of evidence II, grade B).
- Adnexal involvement should be ruled out by pelvic MRI or transvaginal ultrasound (Level of evidence II, B).

### Treatment

The cornerstone of the fertility-sparing treatment for endometrial carcinoma and its precursor endometrial hyperplasia has traditionally been continuous progestin-based therapy. To date, there are no randomised controlled trials comparing the different types of medical treatment in women with endometrial hyperplasia or grade 1 endometrial endometrioid carcinoma.

A meta-analysis assessed the safety and efficacy of the available medical treatment (Piatek et al., 2021). Medroxyprogesterone acetate and megestrol acetate are the most used progestins. Both have been administered orally every day, but dosage varied among studies, while medroxyprogesterone acetate has also been given intra-muscularly twice a week. Megestrol acetate has been shown to result in higher remission rates than medroxyprogesterone acetate and other hormonal treatments, possibly due to its relatively higher bioavailability following oral administration (Lucchini et al., 2021). Patients who received an oral progestin as monotherapy are more likely to experience disease recurrence and more systemic adverse effects. An alternative way of progestin administration is the use of levonorgestrel- intra-uterine device, but its efficacy has not been compared with oral progestins (Floyd et al., 2021). This device in combination with oral progestins or gonadotropin-releasing hormone analogues has been shown to have a satisfactory remission rate and low recurrence rate, with higher cumulative effectiveness compared with the levonorgestrel- intra-uterine device alone (Fang et al., 2021; Gallo et al., 2021). Gonadotropin-releasing hormone analogues show a satisfactory response rate when used alone, and in combination with intra-uterine progestin therapy or oral aromatase inhibitors. In obese patients, gonadotropin-releasing hormone analogues in combination with levonorgestrel-intra- uterine device or oral aromatase inhibitors seem to be preferable (Emons and Grundker, 2021).

Tamoxifen has been evaluated in the treatment of advanced stage and recurrent endometrial carcinoma, giving inconsistent results, so this form of treatment has not been used in early-stage endometrial carcinoma (Kim et al., 2018; van Weelden et al., 2019). Different medical treatment regimens have been described in the literature, including the use of hydroxyprogesterone caproate, norethisterone acetate, natural progesterone, aromatase inhibitors (letrozole, anastrozole), and combined oral contraceptives. There are no comparative studies to determine their efficacy (Peiretti et al., 2019; Zhou et al., 2021). Few studies have evaluated them and no studies assessing efficacy separately are available (Peiretti et al., 2019; Zhou et al., 2021).

Combined treatment with hysteroscopic resection followed by either oral/intra-uterine-released progestins or gonadotropin-releasing hormone analogues appears to be an effective alternative to traditional fertility-sparing treatment in young women with endometrial endometrioid carcinoma and endometrial hyperplasia (Casadio et al., 2019; Falcone et al., 2017; Gallo et al., 2021; Giampaolino et al., 2019). It has been shown to provide certainty on tumour staging as well as myometrial involvement and to allow optimal cytoreduction, facilitating the subsequent therapeutic effect of progestins (Casadio et al., 2019).

**Figure 4:**
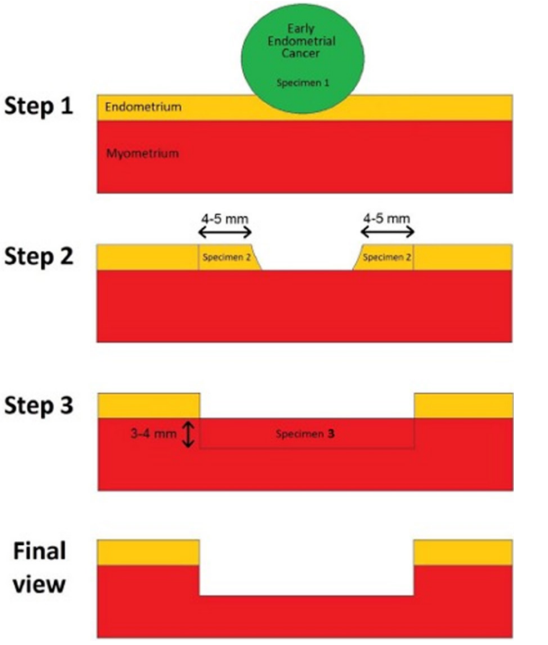
Schematic representation of hysteroscopic resection of focal endometrial endometrioid carcinoma following the ‘three steps’ technique.

Mazzon et al. (2005) first described the three- step hysteroscopic resection of focal endometrial endometrioid carcinoma, consisting of resection of the tumour lesion (step 1), the endometrium adjacent to the lesion (4–5 mm outside) (step 2), and the myometrium underlying the lesion (3–4 mm) (step 3); once the pathology report confirmed grade 1 (G1) endometrial carcinoma without myometrial invasion, then medical therapy with megestrol acetate (160 mg daily) for 6 months was administered (Mazzon et al., 2005) (see Figure 4).

Giampaolino et al. (2019) described a combined fertility-sparing treatment, but they made a distinction between early endometrial carcinoma and endometrial hyperplasia. Patients diagnosed with early endometrial carcinoma underwent hysteroscopic resection following the three-steps technique by Mazzon et al. (2005) adding multiple random endometrial biopsies; a levonorgestrel- intra-uterine device was inserted when the histologic report confirmed early endometrial carcinoma G1 on the lesion, with the surrounding endometrium and the underlying myometrium free of disease. When endometrial hyperplasia is diagnosed, the surgical treatment consisted of superficial endometrial resection, preserving the basal layer of the endometrium, followed by insertion of the levonorgestrel-intra-uterine device right after the procedure (Giampaolino et al., 2019).

A systematic review suggests a higher effectiveness of a high-dose progestins protocol (Piatek et al., 2021). As monotherapy, the dose recommendations for megestrol acetate are 160– 320 mg/day and for medroxyprogesterone acetate 400–600 mg/day (Concin et al., 2021a; Concin et al., 2021b; Concin et al., 2021c). Levonorgestrel at a dose of 52 mg is the only intra-uterine-released progestin ever evaluated.

The exact duration of treatment has not been clearly defined. However, most studies have found a median time to regression of 4 to 6 months. The presence of risk factors, such as obesity and insulin resistance, may require a longer treatment time (Floyd et al., 2021). Therefore, 6–12 months is the recommended duration of therapy within which a complete response should be achieved. If there is no response after 6–12 months, radical surgery is suggested (Gallo et al., 2021). The cut-off point for the duration of treatment for obtaining a complete response has been proposed to be 15 months, and after that time no response has been observed and/ or oncological safety cannot be assessed (Shim et al., 2021).

The aim of conservative treatment is to obtain a complete response, defined as a negative biopsy. The global response rates after conservative medical treatment, with or without previous surgical hysteroscopic excision, in early-stage, low-grade endometrial carcinoma are high, from 75% to 79.4% (Guillon et al., 2019; Li et al., 2019; Lucchini et al., 2021; Peiretti et al., 2019). The significant highest complete response rates are obtained with the combination of hysteroscopic resection followed by progestin treatment, either by oral or by intra-uterine device administration, which varies from 90% to 95.3%. High-dose oral progestins showed a complete response rate of between 76.3% and 77.7%, and levonorgestrel- intra-uterine device with oral progestins between 71.3% and 72.9% (Chen et al., 2016; Falcone et al., 2017; Fan et al., 2018; Garzon et al., 2021; Lucchini et al., 2021; Qin et al., 2016). Partial response rates vary from 4.7% to 7% and the no response rates from 17.2% to 20.9% (Piatek et al., 2021; Roh et al., 2021).

Factors affecting response rates are not completely defined, but they include the molecular profile of the disease, the weight of the patient (improved response rates in patients with a BMI <25 kg/m²), low serum marker HE4 and low histological grade, and polycystic ovarian morphology on ultrasound scan, among others; although there is a lack of evidence on their clinical utility for them to be used routinely (Baxter et al., 2020; Behrouzi et al., 2020; Chung et al., 2021; Fukui et al., 2017; Li et al., 2019).

No randomised controlled trial is available to set a clear and strict interval or assessment method for the follow-up of patients after fertility preservation in endometrial carcinoma. However, since intensive follow-up to assess the endometrial response is needed, most authors recommend endometrial sampling every 3–6 months either by dilation and curettage or by hysteroscopic biopsy (Casadio et al., 2020; Cho et al., 2021; Falcone et al., 2017; Novikova et al., 2021; Zapardiel et al., 2016). The most established and reasonable option for surveillance seems to be a hysteroscopic endometrial biopsy at 3 and 6 months. Two consecutive complete response endometrial biopsies with a minimal interval of 3 months are necessary to consider the success of the fertility-sparing treatment and to recommend pregnancy (Giampaolino et al., 2019). Then, if complete response is achieved, a 3- to 6- month follow-up biopsy is required until pregnancy or until definitive surgery is performed (Gallo et al., 2021). Due to this frequent follow-up, patient agreement is essential for early detection of complete response or relapse after fertility-sparing management (Obermair et al., 2020).

**Figure 5:**
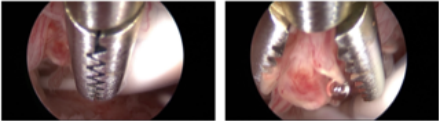
Hysteroscopic endometrial biopsy with the grasp technique, with a levonorgestrel-intra-uterine device in situ (the device should not be removed to perform endometrial biopsy during follow-up. Be careful not to catch the strings of the device in the branches of the grasping forceps, so as not to accidentally remove it).

The correct method of performing a hysteroscopic endometrial biopsy has been described above, but it is to be noted that the levonorgestrel-intra- uterine device should not be removed to perform biopsy (Clark et al., 2002) (Figure 5). In addition, pelvic examination and ultrasound scan might be recommended during the follow-up visits (Cho et al., 2021; Novikova et al., 2021). If recurrent disease is diagnosed during the follow-up, a second attempt at fertility preservation could obtain a complete response, even if the complete response rate is slightly lower than for first treatment (Wang et al., 2019).

Different systematic reviews have suggested the importance of applying assisted reproductive technology to achieve pregnancy in women who have had fertility-sparing treatment for endometrial carcinoma or endometrial hyperplasia, to minimise time prior to definitive surgery and thereby minimise the risk of relapse (Fan et al., 2021; Floyd et al., 2021; Zapardiel et al., 2016; Zhang et al., 2017). Previous studies have shown a higher probability of recurrence when the time to achieve complete response is longer (Koskas et al., 2014). The type of ovarian stimulation and the assisted reproductive technology protocol should be tailored based on the characteristics of each patient, in consultation with a multidisciplinary team, as there is no clear optimal duration, protocol, or number of attempts for ovarian stimulation in these patients. As with stimulation protocols in patients with breast cancer, the use of letrozole with gonadotropins has shown further protection in endometrial carcinoma. (Zapardiel et al., 2016).

There seems to be higher pregnancy rates and live birth rates after fertility-sparing treatments with oral progestins compared with the levonorgestrel- intra-uterine device only. A meta-analysis and systematic review including 28 studies and 1038 patients found a pregnancy rate in the group that received oral progestin of 34% and live birth rate of 20% (Wei et al., 2017). In the groups that received the levonorgestrel-intra-uterine device only, or both levonorgestrel-intra-uterine device and progestin, the pregnancy rates were 18% and 40%, respectively, and live birth rates 14% and 35%, respectively (Wei et al., 2017). Combined treatment, with hysteroscopic resection followed by hormonal therapy, was found to achieve higher live birth rates than with oral progestogen alone (Herrera Cappelletti et al., 2022; Zhang et al., 2017). A meta- analysis including 54 studies found a live birth rate of 53% in the hysteroscopy group compared with 33% in the progestin only group (p=0.09) (Zhang et al., 2017). Certain factors have been associated with a superior pregnancy outcome. In a retrospective study of 68 women with early-stage endometrioid cancer or endometrial hyperplasia, a multivariate analysis revealed that a normal BMI, shorter time to complete remission, a prolonged 3-month treatment, fewer hysteroscopic procedures, and a thicker endometrium were all associated with successful pregnancy (Fan et al., 2021).

There is a lack of studies directly comparing assisted reproductive technology with expectant management in women with endometrial carcinoma and no history of infertility. Younger patients with no known infertility history may attempt a natural pregnancy, as long as close monitoring is provided and within a defined time, encouraging broader use of assisted reproductive technology without significant delay (Novikova et al., 2021; Park et al., 2013; Vaugon et al., 2021). In a prospective study of 232 women with early endometrial carcinoma or endometrial hyperplasia, who attempted conception, 38% used assisted reproductive technology. In contrast to previous data, pregnancy rates as well live birth rates were superior in the natural conception group than in the assisted reproductive technology group (54.7 vs 40.7 and 49% vs 34%, respectively; p=0.04). In that study, women using assisted reproductive technology were significantly older (p=0.03) (Novikova et al., 2021). Therefore, patients would benefit from being referred to a fertility specialist for an early consultation (Kohn et al., 2021). Using assisted reproductive technology shortens the time to conception and avoids prolonged, unopposed oestrogen stimulation, which results in oncological safety and reduction of the risk of relapse and disease progression. No data are reported for obstetrical and neonatal outcomes in babies born to mothers with endometrial carcinoma.

Patients who decline definitive surgery after delivery and those who do not plan their second pregnancy immediately after the first should be recommended to restart maintenance therapy with a levonorgestrel- intra-uterine device (Novikova et al., 2021).

Gunderson et al analysed 45 studies, including 280 patients with G1 endometrial carcinoma treated with progestins (Gunderson et al., 2012). They found a complete response rate of 48% with a median time to response of 6 months; in addition, recurrence rate after complete response was 35% and, finally, persistent, or progressive disease was found in 25% of enrolled subjects. Another meta-analysis including young women with early-stage endometrial carcinoma has shown that a complete response to treatment occurs in about 80% of patients, and the plateau of response occurs after 12 months of progestin treatment. Recurrence occurred in 17% after 12 months and in 29% after 24 months after treatment (Koskas et al., 2014). Qin et al reported more or less similar results with regression rate of 82.4% (95% CI 75.3% to 88.7%) and a relapse rate of 25.0% (95% CI 15.8% to 35.2%)(Qin et al., 2016). Long-term oncological outcomes for hysteroscopic resection have not been adequately studied, but relapse rates in studies of women treated by combined therapy are reported to be lower than those in most recent studies on progestin therapies alone (Falcone et al., 2017). Casadio et al. (2020) carried out the longest follow-up, with a median period of 36 months (range 24–60) and reported a relapse rate of 8.7% in women with endometrial hyperplasia and of 11.11% in women with G1 endometrial carcinoma (Casadio et al., 2020).

Complete response to progestins has been shown to be less frequent among obese than among non- obese patients (4/12 (33%) vs 35/41 (85%); p=0.001), and in patients with a BMI ≥25 kg/m² (p=0.0007, OR=2.5; 95% CI 1.4 to 4.3) (Chen et al., 2016; Li et al., 2019). Furthermore, during the median follow-up of 39 months, 22.3% of the women developed recurrence. One patient (0.09%) died of the disease. Limited evidence indicates that metformin may improve the recurrence risk for patients with BMI ≥25 kg/m² (Mitsuhashi et al., 2019; Mitsuhashi et al., 2016). Although Novikova et al reported that the levonorgestrel-intra-uterine device + gonadotropin- releasing hormone analogues + three dilation and curettage procedures was superior to other treatments (complete response=96%, p=0.026) where two dilation and curettages were performed or oral medroxyprogesterone acetate was prescribed, most other data failed to show a difference in efficacy and recurrence rate between oral progestins and the levonorgestrel-intra-uterine device (Lucchini et al., 2021; Novikova et al., 2021). A meta-analysis showed that hysteroscopic resection followed by progestin therapy led to a complete response and a recurrence rate of 95.3% (95% CI 87.8% to 100%) and 14.1% (95% CI 7.1% to 26.1%), respectively (Fan et al., 2018).

Patients who partially respond to progestin treatment at 6 months may be advised to continue the treatment for an additional 3–6 months, and non- responders at the 6-month follow-up with persistent disease confirmed by biopsy should be counselled about whether to undergo hysterectomy (Obermair et al., 2020).

The indications for post-pregnancy management, failure to conceive, and post-treatment conservative relapse in these patients are still unclear. There are no universally agreed guidelines for this management. All reports are limited to small sample sizes. In the absence of guidelines and unanimous consent, management is entrusted to recommendations, retrospective studies, and reviews.

Definitive surgical treatment consists of total hysterectomy with or without bilateral salpingo- oophorectomy and surgical staging. It should be recommended after completion of childbearing due to a high recurrence rate, in cases of recurrence or no response at 6 to 12 months of hormonal treatment, as well as in cases of disease progression either in the uterus or elsewhere (Concin et al., 2021a; Concin et al., 2021b; Concin et al., 2021c; Floyd et al., 2021; Gallo et al., 2021).

The aim of the definitive surgical treatment is to remove the uterus, where the recurrence most commonly appears. Hence, the a priori removal of ovaries is not warranted (as staging of the disease after primary conservative treatment is not indicated any more). Furthermore, removal of ovaries has no therapeutic effect. A meta-analysis showed that there is no significant difference in overall survival if the ovaries were or were not removed at the time of hysterectomy for early-stage endometrial carcinoma (Gu et al., 2017). Removal of the ovaries should therefore be individualised according to the patient’s age, probability of ovarian involvement, genetic/familiar high risk of primary ovarian cancer, or the presence of adnexal disease. In cases of ovarian preservation, salpingectomy is recommended (Concin et al., 2021a; Concin et al., 2021b; Concin et al., 2021c). The balance between the risks of ovarian cancer versus the consequences of surgical menopause should be considered, and oestrogen replacement after pre-menopausal bilateral salpingo- oophorectomy may be considered. In patients considered to be high risk for surgery or refuse definitive surgery, a second course of conservative treatment (medical therapy or combined treatment) could be performed (Obermair et al., 2020).

As some women may still wish to maintain their reproductive potential despite recurrence, repeating fertility-sparing treatment may be considered (Kalogera et al., 2014; Wang et al., 2019). There are limited reports in the related literature on the efficacy of fertility-preserving re-treatment in patients with relapse, and no consensus has been reached on the treatment of recurrence after fertility preservation. In a single-centre retrospective study, 51 patients were enrolled who had persistent disease (residual carcinoma or endometrial hyperplasia on endometrial biopsy) confirmed by dilation and curettage biopsy after 9 months of progestin-based therapy (Cho et al., 2021). All patients received the same dose and type of progestin as their initial therapy: 72.5% achieved complete response at a median time of 17.3 months; among these patients, 32.4% experienced recurrence. If the disease is progressive, a total hysterectomy with bilateral salpingo-oophorectomy and surgical staging is strongly recommended (Tock et al., 2018).

#### Recommendations

##### Selection of Medication

- A combined approach consisting of hysteroscopic tumour resection, followed by oral progestins and/or levonorgestrel-intra-uterine device, is the most effective fertility-sparing treatment both for complete response rate and live birth rate compared with other treatment options (Level of evidence II, grade B).
- Gonadotropin-releasing hormone analogues should not be considered as a first-line treatment (Level of evidence II, grade B).

##### The Role of Hysteroscopic Resection

- If an early and focal myometrial invasion (1–2 mm) is suspected from the resection material, a fertility-sparing approach may be discussed on a case-by-case basis. In this circumstance, complete hysteroscopic lesion resection, followed by oral progestins and/or levonorgestrel-intra-uterine device, can be proposed as fertility-sparing treatment (Level of evidence IV, grade C).

##### Dose of Progestins

- Orally administered megestrol acetate at a dose of 160–320 mg/day or medroxyprogesterone acetate at a dose of 400–600 mg/day is recommended (Level of evidence III, grade B).
- A levonorgestrel-intra-uterine device at a dose of 52 mg, alone or in combination with oral progestins, is a safe and effective approach (Level of evidence III, grade B).

##### Duration of Treatment

- The recommended duration of therapy is 6–12 months, within which a complete response should be achieved (Level of evidence III, grade B).
- The maximum time to achieve complete response should not exceed 15 months (Level of evidence IV, grade C).
- In the absence of any kind of response at 6 months, multidisciplinary counselling is recommended for adapting the management on a case-by-case basis (Level of evidence IV, grade B).

##### Response (Partial vs Complete vs No Response)

- Hysteroscopic resection followed by progestins either by oral and/or intra-uterine device administration is recommended to achieve both the highest complete response rate and the highest live birth rate (Level of evidence II, grade B).
- Weight control during fertility-sparing treatment is highly recommended to increase the chance of response (Level of evidence II, grade A).

##### Follow-up with Maintenance Treatment for Patients Willing or Not Willing to Conceive Immediately

- Two consecutive endometrial biopsies showing complete response with a minimal interval of 3 months are necessary to consider the success of the fertility-sparing treatment (Level of evidence IV, grade C).
- The complete response is mandatory to consider follow-up with maintenance treatment until pregnancy is planned (Level of evidence II, grade A).
- Clinical pelvic examination and ultrasound scan are recommended at every 3-month follow-up visit (Level of evidence IV, grade B).
- Endometrial histological assessment should be performed every 3–6 months by hysteroscopy according to the results of imaging (Level of evidence IV, grade B).
- MRI could be considered on a case-by-case basis (Level of evidence IV, grade C).

##### Pregnancy

- Women undergoing fertility-sparing treatment for endometrial hyperplasia or endometrial carcinoma should be encouraged to actively aim to conceive as soon as the complete response is achieved (Level of evidence V, grade B).
- Assisted reproductive technology should be considered in order to improve success rate and reduce the interval to conception without a higher risk of recurrence (Level of evidence III, grade B). However, natural conception may be considered in women with good reproductive potential within a defined time (6–9 months) (Level of evidence V, grade C).
- Close surveillance by a multidisciplinary team should be continued and maintenance therapy with a levonorgestrel-intra-uterine device should be recommended to women who decline surgery after delivery and who do not plan their second pregnancy immediately after the first one (Level of evidence III, grade B).

##### Recurrence Rate After Fertility-sparing Treatment

- The risk of recurrence after fertility-sparing treatment for endometrial carcinoma may be equal for progestins or a levonorgestrel-intra-uterine device (Level of evidence II, grade B).

##### Definitive and Completion Surgeries

- Definitive surgery is recommended in cases of non-responders, inability to conceive, recurrence or disease progression (Level of evidence II, grade A).
- For patients with a strong desire to preserve fertility, a second conservative approach can be considered on a case-by-case basis (Level of evidence IV, grade B).
- Completion surgery is recommended after completing childbearing (Level of evidence II, grade A).
- Removal of ovaries should be considered on a case-by-case basis (Level of evidence III, grade B).

### Special issues

Despite the small number of studies available, with evidence not as robust, conservative treatment may be considered in women with early-stage G2 endometrioid adenocarcinoma (stage IA G2 endometrial carcinoma) or with well-differentiated G1 endometrioid adenocarcinoma with minimal myometrial invasion (1–2 mm) (Casadio et al., 2020; Shan et al., 2021). Both these findings were exclusion criteria for conservative treatment in the past. The combined treatment described above, consisting of endometrial hysteroscopic resection followed by either oral/intra-uterine released progestins or gonadotropin-releasing hormone analogues, appears feasible and safe in these women.

A positive oestrogen receptor and progesterone receptor status is associated with a more favourable outcome in the majority of patients with type I endometrial carcinoma (Kleine et al., 1990; Morice et al., 2016). However, their prognostic significance is not universally accepted and remains unclear (Jeon et al., 2006). Zhang et al conducted a systematic review and meta-analysis for the expression rate of oestrogen receptors and progesterone receptors in endometrial carcinoma which included 48 and 38 studies, respectively. (Zhang et al., 2015). They showed that oestrogen receptor and progesterone receptor positivity was an independent favourable prognostic factor for survival.

A meta-analysis of 13 studies, which included 635 patients, showed that oestrogen receptor and progesterone receptor expressions are significantly predictive of response in endometrial hyperplasia and early endometrial carcinoma to conservative treatment using the levonorgestrel-intra-uterine device but not with oral progestins. However, the authors concluded that their accuracy is insufficient to be determined in clinical practice (Raffone et al., 2019).

Hormonal treatment with progestins can be the treatment of choice for young women with endometrial hyperplasia or low-grade endometrial carcinoma who wish to preserve fertility. Yet the complete response and recurrence rates have been reported to range from 66.7% to 79.7% and 19% to 34%, respectively (Chung et al., 2021). Thereafter, incorporating tumour biology into management algorithms might help in developing more accurate risk stratification models to guide treatment. There are insufficient data to support the routine use of several immunohistochemical predictive markers in clinical practice. The pre-treatment immunohistochemical evaluation of oestrogen receptor and progesterone receptor was not found to be accurate in predicting response to treatment, while their expression seems to be influenced by other parameters such as obesity (Busch et al., 2017; Raffone et al., 2019; Travaglino et al., 2019). Research on other molecules reported to be involved in endometrial carcinogenesis, such as PTEN, ARID1A, L1CAM, and β-catenin may prove useful (Ayhan et al., 2015; Hu et al., 2019; Karnezis et al., 2017). Specifically, mutational analysis of CTNNB1 and TP53 might help to identify a subset of patients with low-grade, early- stage endometrial carcinoma who are at higher risk of recurrence, while it was found that the immunohistochemical expression of β-catenin was significantly increased in patients with endometrial carcinoma with progression compared with those without progression after fertility-preserving treatment (Hu et al., 2019; Kurnit et al., 2017).

The ProMisE molecular classifier has shown prognostic significance in endometrial carcinoma, thereby enabling early stratification of clinical trials, referral for hereditary cancer testing, and risk assignment to direct care (Baxter et al., 2020; Britton et al., 2019; Dyer et al., 2021; Kommoss et al., 2018; Stelloo et al., 2016). It can be applied in endometrial biopsy or curettage specimens, with high concordance with hysterectomy material (Britton et al., 2019; Dyer et al., 2021; Kommoss et al., 2018; Stelloo et al., 2016; Talhouk et al., 2017). ProMisE identifies the four Cancer Genome Atlas-based molecular subtypes for endometrial carcinoma by using immunohistochemistry and sequencing for the POLE exonuclease domain (Talhouk et al., 2015). The respective four subgroups are those with mismatch repair-deficient, POLE mutations associated with highly favourable outcomes, and wild-type or aberrant p53 expression (p53wt or p53abn, respectively), the latter associated with aggressive disease. As for the small group of tumours referred to as ‘multiple classifiers’, harbouring more than one molecular classifying feature, specifically those with a mismatch repair- deficient p53abn or POLEmut-p53abn profile, there was supporting evidence to categorise them as single classifier mismatch repair-deficient or POLEmut, since outcomes correspond to those predicted by the driver molecular subtype (Baxter et al., 2020; Leon- Castillo et al., 2020; Soslow et al., 2019). Thereafter all molecular tests should be done in conjunction.

In the younger age group with low-grade, stage IA endometrial carcinomas the greatest benefit of progesterone management is seen in women harbouring p53 wild-type tumours. Since the rare p53abn tumours are more likely to progress, conservative therapy would probably be inappropriate, while for POLE-mutated carcinomas the treatment choice in the conservative era is still unclear (Beinse et al., 2020; Britton et al., 2019; Falcone et al., 2019; Leon-Castillo et al., 2020). As for mismatch repair-deficient tumours, they seem to be usually of higher stage, less responsive to progesterone therapy and highly predictive of recurrence after initial regression (Chung et al., 2021; Puechl et al., 2021; Raffone et al., 2021; Zakhour et al., 2017). Moreover, women with mismatch repair-deficient tumours should be tested for Lynch syndrome since they could be carriers of pathological mismatch repair-deficient gene variants (Ryan et al., 2020; Ryan et al., 2017). If Lynch syndrome is identified, appropriate counselling on the risk of developing additional cancers should be mandatory.

Unfortunately, the number of studies that have evaluated whether ProMisE classification could provide important information on treatment choice for young women with low-grade, low-stage endometrial carcinoma wishing to preserve fertility is limited. Available data now do not show that in the context of low-risk disease the molecular classification adds prognostic value. Large prospective studies are needed to validate its clinical usefulness (Amant et al., 2021; Knez et al., 2021).

#### Recommendations

##### Oestrogen and/or Progesterone Receptors Status

- Oestrogen and progesterone expressions seem to be predictive of response in conservative treatment and could be useful for patient counselling (Level of evidence III, grade C).
- Negative oestrogen and progesterone expressions are not a contraindication for fertility- sparing treatment (Level of evidence III, grade C).

##### Molecular Profiling of Early-onset Endometrial Carcinoma and Correlation with Response to Treatment

- Performing the ProMisE molecular classifier in all young patients with grade 1, low-stage endometrial carcinoma who wish to preserve fertility is encouraged, although available data do not allow clinical applicability (Level of evidence IV, grade B).
- Immunohistochemistry for the identification of mismatch repair-deficient tumours is mandatory in order to identify patients at high risk for Lynch syndrome (Level of evidence III, grade A).
- If a Lynch syndrome is identified, patients should have an appropriate counselling on the risk of developing additional cancers (Level of evidence III, grade A).
- In a tumour with p53abn phenotype, testing for MSH-H and POLE mutation should be considered in order to define whether the tumour belongs to the multiple classifiers or to the copy number high molecular subgroup (Level of evidence III, grade A).
- In women harbouring copy number high (p53abn) tumours, conservative therapy would be inappropriate (Level of evidence IV, grade D).

### What does this mean for patients?

Even if not very common, endometrial carcinoma is diagnosed in pre-menopausal women. The standard treatment for endometrial carcinoma is removal of the uterus and ovaries (total hysterectomy with bilateral salpingo-oophorectomy). While effectively increasing the chances of surviving the disease, this treatment is devastating for young women, who would no longer be able to carry a pregnancy. This paper provides clinical guidance to oncologists and fertility specialists on fertility-sparing treatments in endometrial carcinoma, being medical treatments that can pause further progression of the cancer and allow patients to achieve a pregnancy before the removal of the uterus and ovaries. The guidance includes recommendations for the most effective treatments, and also on how to select patients that could benefit from this approach. Upfront, the guidance recommends that patients with endometrial carcinoma undergoing fertility-sparing treatments are supported by a multidisciplinary team, including at least an oncologist and fertility specialist.
